# Next-Generation Technologies for Multiomics Approaches Including Interactome Sequencing

**DOI:** 10.1155/2015/104209

**Published:** 2015-01-12

**Authors:** Hiroyuki Ohashi, Mai Hasegawa, Kentaro Wakimoto, Etsuko Miyamoto-Sato

**Affiliations:** ^1^Division of Interactome Medical Sciences, The Institute of Medical Science, The University of Tokyo, 4-6-1 Shirokanedai, Minato-ku, Tokyo 108-8639, Japan; ^2^Division of Molecular Biology, Tokyo University of Science Research Institute for Biomedical Science, 2669 Yamazaki, Noda, Chiba 278-0022, Japan

## Abstract

The development of high-speed analytical techniques such as next-generation sequencing and microarrays allows high-throughput analysis of biological information at a low cost. These techniques contribute to medical and bioscience advancements and provide new avenues for scientific research. Here, we outline a variety of new innovative techniques and discuss their use in omics research (e.g., genomics, transcriptomics, metabolomics, proteomics, and interactomics). We also discuss the possible applications of these methods, including an interactome sequencing technology that we developed, in future medical and life science research.

## 1. Introduction

**Figure 1:**
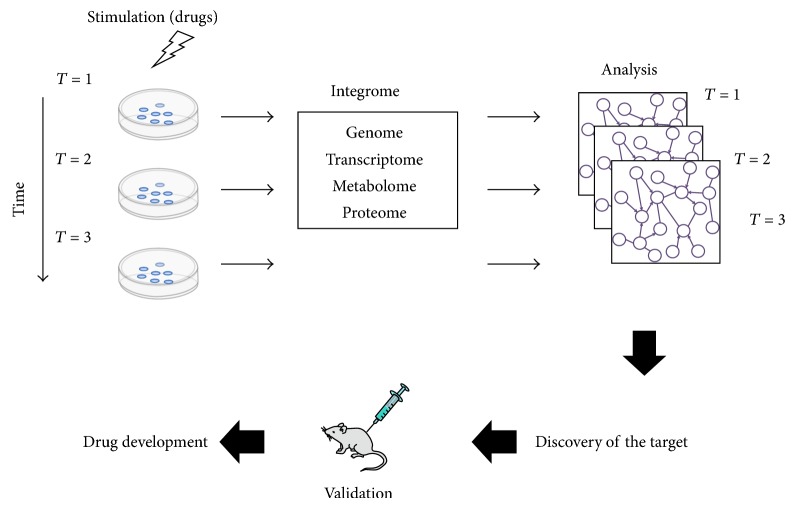
The dynamics of pharmacological response mechanisms can be examined by analyzing integrated multiomics data. First, the time series of the multiomics data are integrated. Second, an efficient module-detecting algorithm is applied to the composite maps. The maps can then be used for comparing cancer cells and normal cells and for assessing the effects of medicines. Lastly, the identified targets can be validated in animal experiments designed for the purpose of subsequent drug development.

More than a decade has passed since the human genome sequence was decoded. Subsequent advancements in and integration of personal genome analysis, post-genome functional analysis, and multiomics analyses have facilitated the development of personalized medicine, which is emerging as the optimal therapeutic direction for the future of medical science (Figure 1). The advent of next-generation sequencing (NGS) and its use in clinical practice will enable the adaptation of multiomics data to personal medical care. However, the costs of these methods and the amount of data generated using multiomics approaches have emerged as challenges that must be tackled. Interactome analysis is considered to be a crucial integrator of multiomics analysis. Currently, the “integrome” is being investigated to determine how the large amounts of data generated using multiomics approaches can be integrated most advantageously. In this review, we discuss these innovative new approaches used in genomics, transcriptomics, metabolomics, and proteomics. We also present an overview of the new insights into complex biological systems that are provided by the use of these technologies.

## 2. Genomics

NGS has contributed substantially to recent advances in omics research. In NGS, a technology used in genome sequencing, sequences containing millions of DNA fragments are read by performing numerous reactions in parallel [1]. The use of this technology has dramatically reduced the time and cost required for sequencing and has facilitated analysis of the human genome, epigenome, and transcriptome. Several NGS platforms have been released by various companies, and a few representative platforms are HiSeq and MiSeq (Illumina), 454 GS FLX (Roche), and PacBio (Pacific Biotechnology) (Table 1). In these platforms, distinct methods of template preparation and signal detection are used [2, 3].

NGS can be used for performing genomic and epigenomic analyses (Table 2). In genomic analysis, somatic mutations are detected using whole-genome sequencing or whole-exome sequencing. In whole-genome sequencing, somatic mutations (e.g., single nucleotide polymorphisms or insertion-deletion mutations) are identified by sequencing the entire genome, and this approach has been used to identify several somatic mutations in various cancers [4, 5].

The use of whole-exome sequencing, which is employed for analyzing exon regions, has identified numerous mutations that occur in disease, such as* BRAF* mutations in papillary craniopharyngiomas [6] and somatic mutations of* BCOR* in myeloid leukemia [7]. Furthermore, this approach has been used for analyzing tumor borders and for detecting the* BRAF* mutation characteristic to borderline tumors. By this approach, 15 novel somatic mutations were detected in serous borderline tumors of the ovary [8]. Thus, genomic analysis performed using NGS provides extensive information about somatic mutations. In addition to genomic analysis, epigenetic analyses are performed using NGS. DNA methylation is involved in transcriptional regulation and it potently affects disease progression. One of the methods used for analyzing the methylation status of DNA (in particular, the methylation of cytosine residues) is bisulfite sequencing. This application was developed based on exploiting the feature that bisulfate treatment converts all residues except methylated cytosine into uracil. The use of bisulfite sequencing has yielded key information regarding the epigenome in the context of cancer and other diseases [9, 10]. Thus, analyzing DNA methylation is critical in the field of epigenetics.

Chromatin immunoprecipitation sequencing (ChIP-seq) can be used for detecting protein binding to target DNA sequences and histone modifications. The method enables analyses of transcription factor binding to gene promoters and epigenetic modifications (e.g., histone modifications) [11, 12]. Moreover, the chromosome conformation capture (3C) method [13, 14] is used for detecting protein-DNA interaction-mediated spatial chromosome proximity, which is involved in transcriptional regulation and coexpression. Studies in which genome-wide 3C methods were employed together with NGS, such as chromosome conformation capture-on-chip (4C) [15], Hi-C [16], and tethered conformation capture (TCC) [17], have shown that the spatial architecture of interphase chromosomes is closely related to DNA-replication timing, activity of genes, and cell differentiation (reviewed in [18]). Chromatin interaction analysis by means of paired-end tag sequencing (ChIA-PET), which is regarded as a combination of ChIP-seq and 3C, has been used for detecting the chromatin organization that is caused by a specific transcription factor [19, 20]. Recently, ChIA-PET studies performed on RNA polymerase II, which is present in the transcription preinitiation complex, comprehensively revealed active promoters, the transcription factors involved in their activation, and the spatial relationships among them [21, 22]. Thus, DNA sequencing has facilitated advances in both genomic and epigenomic analyses.

## 3. Transcriptomics

Similar to the manner in which DNA sequencing analysis has contributed to genomics and epigenomics, RNA sequencing (RNA-seq) has contributed to transcriptome analysis (Table 2). RNA-seq is an RNA-sequencing technology that is mainly used for sequencing mRNAs or long noncoding RNAs (lncRNAs). The mRNA in cells is analyzed in order to quantify gene expression or to detect fusion genes and splice variants in various cancers [23–25]. RNA-seq has also been used for studying gene expression patterns unique to certain cancers, including lung and renal carcinomas, and this has enabled researchers to identify novel biomarkers for specific types of cancer [24, 26, 27]. Microarrays are also used for analyzing gene expression, but RNA-seq differs from that approach in the following manner: using RNA-seq, absolute quantification of expression is performed, whereas, using microarrays, relative expression is calculated. Moreover, an additional advantage of RNA-seq is that it can be used for detecting unknown transcription products, whereas microarrays cannot be used for this purpose. Certain transcription products have been detected using RNA-seq, and, in a few recent studies, RNA-seq was used for analyzing lncRNAs.

Thus, advances in this field of study have been made possible by the use of NGS-mediated analysis of the transcriptome. NGS has also already been successfully used for detecting mutations in cancer genes, and future research is expected to identify more of these mutations, which might be of therapeutic value.

## 4. Metabolomics

Metabolomics differs from nucleic acid-based-omics methods. Using metabolomics approaches, metabolites contained in a sample can be detected and their concentrations can be determined. This strategy is based on the premise that differences in metabolites reflect differences in biological processes.

Shifts in metabolite composition and changes at the genetic level enable the screening of potential biomarker candidates or therapeutic targets. For instance, high levels of reactive carbonyl compounds and low levels of vitamin B_6_ are observed in the plasma of patients with certain subtypes of schizophrenia [28], suggesting that the use of the carbonyl-scavenger pyridoxamine might provide therapeutic benefits for these patients [29]. The relationship between cancer and changes in metabolites is widely recognized. For example, the Warburg effect describes the process whereby cancer cells preferably use the glycolytic pathway to produce ATP, even when sufficient oxygen is present [30]. The recent accumulation of knowledge based on metabolomics could enable advances in early cancer detection. For example, metabolomics studies have revealed that the profile of free amino acids in plasma is altered in the presence of cancer [31]. This information might lead to the development of novel metabolomics-based screening for early detection of a malignancy.

Most methods used in metabolomics involve separation and detection processes (Table 3) [32–35]. Researchers have typically relied on chromatography—gas chromatography (GC) and high-performance liquid chromatography (HPLC)—and capillary electrophoresis (CE) for separation, whereas they have used nuclear magnetic resonance (NMR) or mass spectrometry (MS) for detection [36, 37]. However, the drawbacks of these approaches have led researchers to combine two or more methods (e.g., liquid chromatography and MS (LC-MS/MS) plus NMR) in metabolomics studies [38].

Metabolomics is divided roughly into two categories based on the experimental methods used: nontargeted and target-defined metabolomics. Nontargeted metabolome analysis is extremely attractive because this method can be used to identify an unknown metabolite and to concurrently determine its relative amount; thus, this method is suitable for nonbiased metabolite fingerprinting and diagnostic-marker exploration. However, nontargeted analysis performed using a single routine method remains challenging. This is because the metabolome includes compounds that differ considerably in molecular weight, electric charge, and concentration. Furthermore, although reference mass-spectrum databases [39–43] have grown rapidly and NMR microassays [44] have been improved, the molecular structures of unknown compounds present in trace amounts cannot be readily determined. Conversely, in several cases, identifying and determining the concentrations of all metabolites is not necessary. For example, only approximately 3,000 types of compounds are currently recognized in relation to human disease [45]. Because MS/MS can be used for detecting hundreds of compounds present in a single extract [46], targeted analysis is more suitable than nontargeted analysis for certain types of application, such as when the research is focused on a specific metabolic pathway. Therefore, future analyses are likely to involve the use of wide-targeted approaches in which several targeted experiments are combined when target compounds are available.

## 5. Proteomics

Comprehensive proteome analysis includes expression proteomics and interaction proteomics. Expression proteomics reveals protein expression patterns in cells, and this approach has been typically used for analyzing the expression status of various proteins by using two-dimensional electrophoresis (differential display) and MS [47]. However, proteins typically do not act alone and must interact with other proteins in order to perform functions. Therefore, interactions between proteins should be analyzed comprehensively. Comprehensive analysis of protein-protein interactions (PPIs) is critical in the fields of proteomics, functional genomics, and systems biology. PPIs are detected using methods that can be divided into* in vivo* and* in vitro* techniques (Table 4). Among these methods,* in vitro* virus (IVV) and yeast two-hybrid (Y2H) tests allow the application of interactome sequencing (Table 2).

### 5.1. *In Vivo* Methods

The Y2H assay is an* in vivo* approach used to detect PPIs [48]. The assay requires two protein domains: a DNA-binding domain and an activation domain that is involved in the activation of DNA transcription. These domains are necessary for the transcription of reporter genes [49, 50]. The Y2H assay allows PPIs to be directly recognized, although in the analysis performed using this method false-positive interactions can appear. The data generated using* in vivo* techniques contain extremely high levels of false positives and false negatives. For example, although the exact rate of false-positive results in Y2H experiments is unknown, early estimates were that these are as high as 70% [51]. False-positive rates in AP experiments could be as high as 77% [52].

### 5.2. *In Vitro* Methods

Traditionally, PPIs* in vitro* have been detected using the tandem affinity purification (TAP) method [53, 54]. In this method, a bait containing tags allows protein complexes to be purified. The purified complexes are identified using a separate approach, such as MS [53, 54]. TAP tags have been developed which can be used for studying complex* in vivo* PPIs without the requirement of prior knowledge [53]. The TAP tagging technology has been used for analyzing the interactome in yeast [55]. The method is based on the attachment of two tags to a protein of interest, which is followed by a two-step purification process [56]. Proteins complexed with the target protein can then be identified using MS [57] and sodium dodecyl sulfate-polyacrylamide gel electrophoresis. The main advantage of TAP-MS is that it can be used for analyzing PPIs comprehensively in the form of protein complexes (which the target protein would be a part of* in vivo*) through the identification of a wide variety of protein complexes [56].

The protein microarray method is currently being established as a powerful tool for detecting proteins, observing their expression levels, and probing their interactions and functions. A protein microarray is a glass plate on which single proteins are bound at distinct positions according to a defined procedure [58]. Protein microarrays have been developed in order to allow an operator to process multiple samples in parallel by using an automated process; this enables efficient and sensitive high-throughput protein analysis.

Another method that allows high-throughput identification of PPIs in original extracts is shotgun proteomics [59]. In this technique, proteins are digested with a protease immediately after extraction and then the resulting peptides are separated using LC; subsequently, the amino acid sequences of the peptides are determined using MS/MS.

The development of the techniques described thus far has facilitated the high-throughput identification of protein interactions. However, the number of proteins that cannot be detected or identified even using these methods, such as the proteins that are expressed at extremely low levels or are highly insoluble, is considerably greater than the number of proteins that can be detected or identified (e.g., abundant, soluble proteins). Therefore, new technologies are required for performing highly efficient high-throughput analysis of numerous proteins.

**Figure 2:**
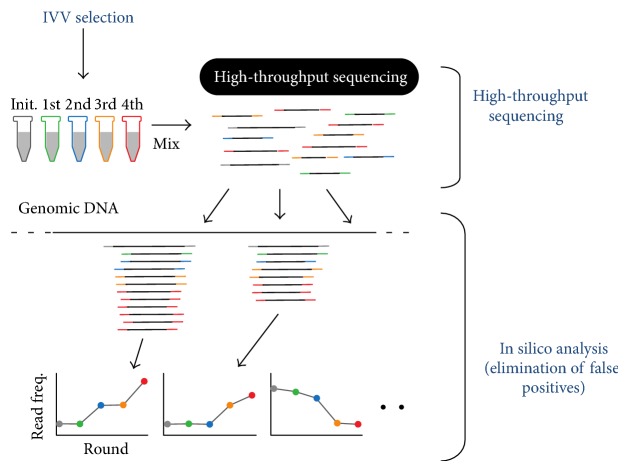
The type of primer used contains a barcoded region (indicated in grey, green, blue, yellow, and red), with four selection-round-specific bases. The reads generated using high-throughput sequencing are sorted according to their barcoded parts and mapped to known genomic sequences. Read frequencies of each genomic position are calculated for each selection round and used for determining the enriched regions. Using barcoded primers can reduce the risk of cross-contamination between libraries. Moreover, in a PPI analysis, increasing the sequencing depth can help detect contamination between samples. In the experiment shown, the Roche 454 Sequencer was used. Statistical significance was calculated by comparing the read frequencies with the frequencies of the initial library and the negative control.

The IVV-high-throughput sequencing (IVV-HiTSeq, Figure 2) method [60], which is a combination of NGS and IVV, has been developed with the aim of overcoming the aforementioned challenges. In the IVV method, protein interactions are selected under cell-free conditions [61–65], and the subsequent sequencing by means of NGS is not limited by cloning steps performed using any specific type of cell. Thus, using the IVV-HiTSeq method, large amounts of accurate protein-interaction data can potentially be generated. The cell-free aspect of the experimental procedure is one of the main advantages: it allows highly efficient production of interaction data. When IVV and HiTSeq are combined, no host cells are required for the purpose of DNA cloning, a step that previously limited the efficiency of screening and the number of interactions that could be examined. Furthermore, the IVV method can be used to select proteins from a cDNA library consisting of 10^12^ molecules, which is beyond the capacity of conventional high-throughput protein-selection methods [66, 67]. The coverage of the interactome is expected to increase in line with the further increases expected in the NGS throughput. Importantly, this completely cell-free procedure will also allow cytotoxic proteins to be analyzed, which will make interactome analysis more comprehensive than it currently is. With respect to the accuracy of IVV-HiTSeq data, the use of library-specific barcoded primers and* in silico* analysis reduces the number of false-positive interactions contained in the initial raw data [60]. IVV-HiTSeq was compared with conventional IVV performed using Sanger sequencing for the same prey library and bait. Whereas 640 sequences (87%) determined using Sanger sequencing were also obtained using IVV-HiTSeq, most of the sequences (99.7%) obtained using IVV-HiTSeq were new and were not detected using Sanger sequencing. Moreover, 88% of the real-time polymerase chain reaction (PCR) assays performed and followed up with the use of IVV-HiTSeq, which included* in silico* analyses, were positive. IVV-HiTSeq can potentially be applied to several cell-free display technologies, such as mRNA display, DNA display, and ribosome display. Moreover, the IVV test can be applied not only to* in vitro* selection of PPIs but also to the detection of protein-DNA, protein-RNA, and protein-chemical compound interactions [68]; this suggests that IVV-HiTSeq could become a universal tool for exploring protein sequences and interaction networks [47].

## 6. Discussion

To further our understanding of living cells, we must collect data by using multiomics analysis. Multiomics includes gene-, transcription-, metabolite-, and protein-specific information. By contrast, the interactome includes network data that are obtained based on direct interactions between molecules. Therefore, an integrated approach, which includes both interactome and multiomics data, is required for comparing the identities of individual cells. This approach is referred to as “integrome” analysis [69]. The integrome is a network map of the interactome together with a list of multiomics data that allows analyses of differences between cancer cells and normal cells [70], the effects of treatments, and key factors such as biomarkers. Because PPIs lie at the core of biomolecular networks, an IVV system designed for detecting PPIs has been developed in an attempt to work toward personal genomics [71], and, using this system, noteworthy results of interactome analysis have been obtained. An advantage of IVV is the large library size (up to 10^12^) that can be analyzed. This large size of the library increases the probability of selecting extremely rare sequences, and it also enhances the diversity of the selected sequences.

The use of NGS will allow the potential of IVV to be maximized. The latest Roche 454 Sequencer can sequence approximately 10^6^ reads, at a rate of approximately 1000 bp per run. This capacity is sufficient for covering both of the linked variable regions. Although 10^6^ reads are not adequate for covering the entire selected IVV library, this advance in NGS might make it possible to obtain unique, high-affinity binders.

Improvements in NGS might also facilitate the development of additional applications, and particularly notable is the speed with which NGS technology is being improved. When we have the capability to sequence the entire selected IVV library, we should be able to select low-affinity ligands that are commonly lost in a typical selection of repetitious rounds. IVV libraries will be subjected to high-throughput sequencing performed using NGS in order to generate interactome information, which will facilitate the archiving of the interactome map of a whole-cell library at a low cost. The use of IVV systems could make key contributions to our understanding of the interactome networks in cells and thus help in the development of pharmaceutical agents for treating currently intractable diseases [71].

## Figures and Tables

**Table 1:** Comparison of representative NGS platforms.

Platform	Company	Detection	Run time	Read length (bp)
454 GS FLX Titanium XL+	Roche	Pyrosequencing	23 hours	700
454 GS Junior System	Roche	Pyrosequencing	10 hours	400
HiSeq 2000/2500	Illumina	Fluorescence	12 days	2 × 100
MiSeq	Illumina	Fluorescence	65 hours	2 × 300
Ion torrent	Life Technologies	Proton release	3 hours	35–400
Ion Proton	Life Technologies	Proton release	4 hours	125
Abi/solid	Life Technologies	Fluorescence	10 days	50
PacBio RSII	Pacific Bioscience	Fluorescence	2 days	−8500

**Table 2:** Types and features of next-generation sequencing technologies.

Type of analysis	Type of sequencing	Feature
Genome	Whole-genome sequencing	Used to detect somatic mutations by sequencing the whole genome
	Whole-exome sequencing	Used to detect somatic mutations by sequencing the whole exon region

Epigenome	Bisulfite sequencing	Used for analyzing methylation by sequencing genome exhaustively
	ChIP-seq	Used to detect the targets of transcription factors or analysis of histone modifications
	DNase-seq	Used for analysis of chromatin architecture
	FAIRE-seq	
	Hi-C	
	ChIA-PET	Used to characterize chromatin interactions that are mediated by nuclear protein of interest

Transcriptome	RNA sequencing	Used for analysis of gene expression or detection of fusion genes and splice variants

Interactome	IVV-HiTSeq	Used to detect reliable protein (domain) interactome without cloning including interactions of protein-protein/DNA/RNA/metabolic compounds/small molecules/drugs and so forth, suitable for high-throughput application, acquisition of high-reliability datasets, and analysis of cytotoxic proteins
	Y2H-seq	Used to detect interacting proteins or protein-domain pairs, but mating and the following diploid culture become the rate-limiting steps when applied in high-throughput technologies

ChIP-seq: chromatin immunoprecipitation sequencing; FAIRE-seq: formaldehyde-assisted isolation of regulatory elements sequencing; ChIA-PET: chromatin interaction analysis by means of paired-end tag sequencing; IVV-HiTSeq: IVV high-throughput sequencing; Y2H-seq: yeast two-hybrid interaction screening approach involving short-read second-generation sequencing.

**Table 3:** Comparison of major metabolomics methods.

Method	Benefit	Drawback
NMR	(1) Results obtained in a single experiment (2) Fast (3) Available for studying various nuclei (4) Consistent with liquid and solid matrices (5) Quantitative (6) Sample can be recovered after analysis	(1) Requires highly skilled technicians and statisticians (2) Limited sensitivity (1–10 µmol/L) (3) Coresonant metabolites can be challenging to quantify

GC-MS	(1) High accuracy and repeatability of results (2) Small amounts of samples are required (3) High discrimination between molecules exhibiting very similar structures (4) High sensitivity (1 pmol/L)	(1) Sample preparation (including derivatization) can be time-consuming (2) Not all compounds are suitable for gas chromatography

LC-MS	(1) Wide application (2) High sensitivity (1 pmol/L)	(1) Requires extensive sample preparation, including derivatization (2) Long analytical time (20–60 min per sample) (3) Limited to volatile compounds (4) Suffers from ion suppression

CE-MS	(1) Suitable for polar molecules (2) Large separation capacity (3) High sensitivity (1 pmol/L)	(1) Low repeatability

**Table 4:** Comparison of comprehensive protein-protein interaction analysis methods.

Method	Selection condition of PPIs	Library size	Cell cloning required	Next-generation sequencing
Y2H	*In vivo *	10^6^	Yes	Applicable but limited
TAP-MS	*In vitro *	Living body sample	Yes	Inapplicable
Protein microarray	*In vitro *	Living body sample	No	Inapplicable
Shotgun proteomics	*In vitro *	Living body sample	No	Inapplicable
IVV	*In vitro *	10^12^	No	Applicable and effective

See also Table 1.

Y2H: yeast two-hybrid; TAP-MS: tandem affinity purification-mass spectrometry; IVV: *in vitro* virus.
